# Adult ADHD clinics in north Wales - case load prevalance & compliance with nice guidelines (quality evaluation project)

**DOI:** 10.1192/bjo.2021.899

**Published:** 2021-06-18

**Authors:** Jawad Raja, Zeenish Azhar, Alberto Salmoiraghi

**Affiliations:** 1 ST6 GA Trainee Betsi Cadwaladr University Health Board; 2 CT3 Trainee Betsi Cadwaladr University Health Board; 3 Medical Director Betsi Cadwaladr University Health Board

## Abstract

**Aims:**

Measure compliance with National Institute for Health and Care Excellence (NICE) recommendations in four Adult CMHT's

Guide further service development.

We audited the case notes of 20 patients each currently under care of 9 General Adult Consultants across 6 CMHT's in East side of North Wales against NICE standards using an adapted version of the ADHD audit support tool.

My role in the Project & How does this represent my practice?

I was the audit and overall lead for this project

I formulated the audit tool and registered my project with Audit Registration Team.

I lead data collection and compilation of results.

**Method:**

Overall, this is the first audit of Adult ADHD Services in East side of North Wales.

It established good compliance with NICE guidance for assessment and treatment.

NICE has expressed the need for full mental health and social assessment including full history and physical examination prior to the drug treatment.

Good compliance was observed in using & documenting Diagnostic Criteria (DSM-IV and/or ICD-10).

There were deficiencies in conducting or arranging recommended physical examination & side effect monitoring.

Drug treatment was the first line of treatment in the majority of cases.

Antipsychotics were used in some patients referred for ADHD assessment, despite the fact that NICE has ruled out the use of antipsychotic drugs in treatment of core symptoms of ADHD.

**Result:**

Prevalence of Adult ADHD clinician case load in Wrexham and Fintshire Counties.

Diagnosis of Adult ADHD according to ICD 10 & DSM IV Guidelines.

Pre treatment screening of physical health for ADHD patients.

Side effects monitoring of patients on stimulant medications.

**Conclusion:**

The finding highlights the need for more effort in educating clinicians about safety and effectiveness of antipsychotics in ADHD.

Comprehensive treatment programmes that address psychological, behavioural, educational and occupational needs should be established.

Development of local ADHD Clinics, support groups and in partnership with the voluntary sector should be encouraged.

It is important that mental health professionals receive appropriate training in assessment, management & monitoring of ADHD patients with co morbid substance use disorder and other mental illnesses.

BETSI Health Board to participate in national Prescribing Observatory for Mental Health (POMH-UK) Quality Improvement Programme (QIP) focusing on prescribing for ADHD in children, adolescents & adults.

